# Prevalence of multidrug-resistant organisms in refugee patients admitted to a German university hospital depending on duration of stay in Germany

**DOI:** 10.3205/dgkh000323

**Published:** 2019-06-28

**Authors:** Claudia Reinheimer, Parisa Abdollahi, Kai Zacharowski, Patrick Meybohm, Haitham Mutlak, Thomas Klingebiel, Thomas A. Wichelhaus, Volkhard A. J. Kempf

**Affiliations:** 1 Institute of Medical Microbiology and Infection Control, University Hospital Frankfurt, Germany; 2 University Center of Infectious Diseases, University Hospital Frankfurt, Germany; 3 University Center of Competence for Infection Control of the State of Hesse, Frankfurt Main, Germany; 4 Department of Anesthesiology, Intensive Care Medicine and Pain Therapy, University Hospital Frankfurt, Germany; 5 Department of Pediatric and Adolescent Medicine, University Hospital Frankfurt, Germany

**Keywords:** refugee, multidrug-resistant organisms, infection control

## Abstract

**Background:** Refugees have a significant risk of carrying multidrug-resistant organisms (MDRO), including multidrug-resistant gram-negative organisms (MDRGN) and methicillin-resistant *Staphylococcus aureus* (MRSA). Since the duration of MDRGN colonization has been shown to last for several months, we hypothesize that the prevalence of MDRO in refugees gradually declines during their stay in Germany to the level of MDRO prevalence in non-refugee patients. Knowledge about the dynamics of refugees’ MDRO prevalence might provide the basis for appropriate infection control measures for refugees in hospitals as well as refugees’ MDRO epidemiology in general.

**Material** **and** **methods:** MDRO prevalence in 109 refugees admitted to the University Hospital Frankfurt, Germany, were compared to 819 adult controls and 224 pediatric patients admitted to the intensive care unit between June 2016 and May 2017.

**Results:** 41.3% (95% confidence interval=31.9–51.1) of the refugees, 5.7% (4.2–7.6) of the adult controls and 8.9% (5.5–13.5) of the pediatric controls were positive for at least one MDRGN. The highest MDRGN prevalence was found in refugees who recently arrived (≤3 months) in Germany (72.4%; 52.8–87.3). Refugees’ MDRGN prevalence declined continuously over time, reaching the adult and pediatric controls’ MDRGN prevalence 18 months at the earliest after their arrival in Germany, i.e., 14.9% (1.8–42.8).

**Conclusion:** This study demonstrates that refugees’ MDRGN prevalence is declining over time since their arrival in Germany. 18 months after their arrival, refugees’ and locals’ MDRGN prevalence no longer differs significantly, although the refugees’ MDRGN prevalence is still higher. A decline of MRSA prevalence was found 18 months after refugees’ arrival. However, MRSA prevalence was still 14%, and thus 8 times higher than that of controls, indicating that precautionary measures continue to be necessary to prevent MRSA transmission.

## Zusammenfassung

**Hintergrund:** Für Flüchtlinge ist ein erhöhtes Risiko bekannt, Träger multiresistenter Organismen, v.a. multiresistenter Gram-negativer Organismen (MRGN) oder Methicillin-resistenter *Staphylococcus aureus* (MRSA), zu sein. Vor dem Hintergrund, dass für MRGN eine Nachweisdauer von mehreren Monaten beschrieben ist, haben wir vermutet, dass sich die MRGN-Prävalenz bei Flüchtlingen in Abhängigkeit von deren Aufenthaltsdauer in Deutschland allmählich der der einheimischen Bevölkerung angleicht. Kenntnisse um die Dynamik der MRGN-Prävalenz bei Flüchtlingen können dazu beitragen, krankenhaushygienische Maßnahmen situationsgerecht anzupassen.

**Material** **und** **Methode** Retrospektive Evaluierung der MDRO-Prävalenz bei 109 Patienten mit Flüchtlingsanamnese im Vergleich zu 819 erwachsenen sowie 224 pädiatrischen Intensivpatienten, die zwischen Juni 2016 und Mai 2017 am Universitätsklinikum Frankfurt behandelt wurden.

**Ergebnisse:** Bei 41,3% (95%-Konfidenzintervall: 31,9–51,1) der Flüchtlinge, 5,7% (4,2–7,6) der erwachsenen und 8,9% (5,5–13,5) der pädiatrischen Intensivpatienten lässt sich mindestens eine MRGN-Spezies nachweisen. Die höchste MRGN-Prävalenz findet sich bei kürzlich (≤3 Monate) in Deutschland eingereisten Flüchtlingen (72,4%; 52,8–87,3). Die MRGN-Prävalenz bei Flüchtlingen mit einer Aufenthaltsdauer >18 Monate unterscheidet sich nicht mehr signifikant von der der Kontrollgruppen (14,9%; 1,8–42,8).

**Schlussfolgerung:** Diese Studie untersucht den Zusammenhang zwischen der Aufenthaltsdauer der Flüchtlinge in Deutschland und deren MRGN-Prävalenz. Es zeigt sich, dass die MRGN-Prävalenz bei Flüchtlingen umso niedriger ist, je länger sie sich in Deutschland aufhielten. Bei Flüchtlingen mit einer Aufenthaltsdauer von mindestens 18 Monaten findet sich im Vergleich zu den hier untersuchten Kontrollgruppen kein statistisch signifikanter Unterschied mehr, wenngleich auch nach dieser Zeitspanne die MRGN-Prävalenz bei Flüchtlingen noch über der Prävalenz der einheimischen kritisch-kranken Kontrollkohorte liegt. Hinsichtlich MRSA zeigt sich bei der Gruppe der Flüchtlinge ebenfalls eine Abnahme der Prävalenz über Zeit. Da allerdings die MRSA-Prävalenz in dieser Patientengruppe auch 18 Monate nach Einreise in Deutschland die der einheimischen Bevölkerung nahezu achtfach übersteigt, sollten risikoadaptierte Hygienemaßnahmen zur Vermeidung einer MRSA-Übertragung auch nach dieser Zeit strikt eingehalten werden.

## Introduction

Refugee patients’ countries of origin have previously been described as high-prevalence countries for multidrug-resistant organisms (MDRO), including multidrug-resistant Gram-negative bacteria (MDRGN) and methicillin-resistant *Staphylococcus* *aureus* (MRSA) [1], [2], [3], [4], [5], [6], [7], [8]. Furthermore, refugee patients have a significant and inherent risk of carrying MDRGN and/or MRSA [1], [2], [3], [4], [5], [6], [7], [8]. Therefore, the need for stringent infection control management for refugee patients, e.g., screening procedures for MDRO, has clearly been proven for German hospitals [1], [2], [3], [4], [5]. In June 2015, an infection control management concept for refugee patients was implemented at University Hospital Frankfurt, Germany [1]: refugee patients are screened on the day of admission and are preemptively isolated until negative results for both MRSA and MDRGN are available. In case of testing positive for MRSA and/or MDRGN, patients remain in isolation during their entire stay at University Hospital Frankfurt, Germany. This infection control procedure aims to guarantee best medical practice both for refugee and all other patients. 

However, it has yet to be determined whether the MDRO prevalence in refugee patients depends on the time elapsed since their arrival in Germany. Evidence for such a time-dependent risk may be provided by O’Fallon et al., who showed that the median duration of MDRGN colonization was 144 days, ranging from 41 to 349 days [9]. In turn, this knowledge might contribute to the discussion on appropriate infection control management of refugee patients and of MDRGN-colonized patients in general.

## Methods

### Infection control surveillance

According to German infection protection law, it is mandatory for hospitals to implement a documented infection control strategy in order to prevent the transmission of infective agents and the health threat that might arise from transmission. In order to fulfill this requirement, German medical institutions are obliged to follow the recommendations by the Commission for Hospital Hygiene and Infection Prevention (KRINKO) at the Robert Koch Institute, Berlin, Germany (RKI), e.g. the “Recommendations for prevention and control of MRSA in medical and nursing facilities” [10] and “Hygiene measures for infection or colonization with multidrug-resistant Gram-negative bacilli” [11]. The University Hospital Frankfurt, Germany (UHF) carefully adheres to these and other recommendations. The high number of international patients admitted to UHF, which might partly be due to the proximity to Frankfurt International Airport, requires an additional, situation-adapted infection control procedure at UHF. Therefore, all patients who were in hospitals in a high-MDRO country and are now seeking admission to UHF, or arriving from refugee accommodations, are pre-emptively isolated and screened for MDRO on the day of admission. Screening procedures are also applied to all patients admitted to any intensive care unit. Immediately after negative results for MDRGN and MRSA are available, patients are released from isolation. In the case of any positive result for MRSA and/or MDRGN with carbapenem resistance, patients remain isolated during their entire stay to prevent MDRO transmission, as previously described [1]. Furthermore, in certain wards with increased infection control demands (e.g., neonatal patients, bone marrow transplantation or burn injuries), isolation also applies to patients testing positive for MDRGN due to (ESBL) phenotype, with additional resistance to fluoroquinolones. These procedures are in accordance with guidelines of infection prevention commission (KRINKO) at the Robert Koch Institute, Berlin, Germany [10], [11].

### Patients and specimens

We retrospectively evaluated the data of 109 patients admitted from refugee accommodations (REF) to the UHF between June 15^th^ 2016 and May 15^th^ 2017. These data were compared to data collected from 819 control non-refugee patients admitted to the intensive care unit (ICU) of the Department of Anesthesiology, Intensive Care Medicine, and Pain Therapy between June 15^th ^2016 and May 15^th^ 2017. Additionally, we determined the MDRO prevalence in 224 critically ill pediatric patients admitted to the Department of Pediatric and Adolescent Medicine, University Hospital Frankfurt, Germany within the same period. For both control patient groups, the assumption was made that these most likely reflect the critically ill adult and pediatric patients’ MDRO prevalence in the Rhine-Main region. As indicated above, patients admitted to the ICU underwent mandatory screening for MDRO on the day of admission. All patients were systematically screened via rectal and nasopharyngeal swabs for MDRO. including MRSA (nasopharyngeal swab, in accordance to [10]) and MDRGN (rectal swab). Swabs were obtained by the respective ward’s staff. Transportation to and storage in the Institute of Medical Microbiology and Infection Control were performed in accordance to the manufacturer’s recommendations as well as the Standards of Quality of Microbiological and Infectiological Diagnostics (Qualitätsstandards in der mikrobiologischen und infektiologischen Diagnostik), in particular chapters 20–23 [12], [13], [14], [15].

MDRGN are defined as Enterobacteriaceae with extended-spectrum beta-lactamase (ESBL) phenotype as well as Enterobacteriaceae, *Pseudomonas aeruginosa*, and *Acinetobacter* *baumannii* resistant to Piperacillin, any 3^rd^/4^th^ generation cephalosporin, and fluoroquinolones. MDRGN with additional resistance to carbapenems (CR) are assigned to “MDRGN with CR”. This approach has been described previously [1], [2]. 

Patients admitted from a refugee accommodation to UHF were assigned to the group “refugee patients”. However, due to language difficulties in patients’ anamnesis, data regarding country of origin, route taken to reach Germany, duration in Germany, residential status (living in small facilities or in mass camps) etc. were not or not completely available in some cases. In contrast to a longitudinal study, follow-up data were not available for refugee patients due to this population’s mobility within Germany (e.g., relocation from a reception center to a facility elsewhere). 

In order to exclude any effects of nosocomially acquired MDRGN affecting MDRGN prevalence, adult patients who were screened prior to admission to the ICU were excluded from this study. In comparison to the adult group, the pediatric group is by nature fewer in number, wherefore we decided not to exclude any of the latter. With regard to age, the refugee group is more similar to the adult group than the pediatric group, which could legitimize this approach.

In terms of infection control, refugee patients with unknown duration of stay since arrival in Germany also need management in the hospital, and were therefore not excluded from this investigation.

### Data regarding country of origin and refugees’ duration of stay in Germany 

We examined the patients’ digital data files regarding aspects of residential status in a refugee accommodation and history of refugee status.

### Definition of time-frames 

In order to assess the MDRO prevalence in refugee patients according to their reported time of arrival in Germany, we defined the following five time-frames for refugees’ duration of stay in Germany:

1. ≤3 months,
2. >3 to <6 months,
3. ≥6 to <12 months,
4. ≥12 to <18 months,
5. ≥18 months.

As peak migration to Germany was almost 18 months ago [16], the longest time-frame was defined as ≥18 months. Since refugee patients have furthermore been suggested to probably represent the general prevalence of MDRO in their country of origin [2], we investigated the prevalence of MDRO in recently arrived refugee patients and therefore set the shortest time-frame to ≤3 months. Furthermore, the median duration of MDRGN colonization has recently been shown to be 144 days, ranging from 41 to 349 days [9]. Due to this finding, we set further time-frames to 6 months and 12 months.

### Detection of MDRGN and molecular resistance analysis

Laboratory testing was performed under strict quality-controlled criteria (laboratory accreditation according to ISO 15189:2007 standards; certificate number D-ML-13102-01-00, valid through January 25^th^, 2021) at the Institute for Medical Microbiology and Infection Control, University Hospital Frankfurt, Germany. Rectal swabs were collected using culture swabs with Amies collection and transport medium (Hain Lifescience, Nehren, Germany), and streaked onto selective CHROMagarTM ESBL plates (Mast Diagnostica, Paris, France). Presumed MDRGN species were identified by matrix-assisted laser desorption ionization time-of-flight analysis (MALDI-TOF) and VITEK 2 (bioMérieux, Nürtingen, Germany). Antibiotic susceptibility testing was performed according to the Clinical and Laboratory Standards Institute (CLSI) guidelines using VITEK 2 and antibiotic gradient testing (bioMérieux). Carbapenemase encoding genes were detected with PCR analysis and sequencing was subsequently performed from carbapenem-resistant Enterobacteriaceae, including the *bla* genes for carbapenemases NDM, VIM, IMP, OXA-48-like and KPC as well as OXA-23, OXA-24, and OXA-58 for *A. baumannii* [17], [18].

### Detection of MRSA and determination of spa type

For the detection of MRSA, nasal swabs were inoculated on Brilliance MRSA Agar (Oxoid, Wesel, Germany). MRSA was identified by MALDI-TOF, and antibiotic susceptibility was tested according to CLSI guidelines using VITEK 2. Clonal identity of MRSA isolates was analyzed by staphylococcal protein A *(spa)* typing using Ridom StaphType software (Ridom GmbH, Würzburg, Germany) [19].

### Statistical analysis

The chi-squared test was performed for statistical analysis. 95% confidence intervals (95% CI) for frequencies were calculated based on binomial distribution and used to confirm statistical significance. *P*-values (2-tailed) of p≤0.05 were considered statistically significant.

### Ethics approval 

This study was approved by the Ethics Board of the University Hospital Frankfurt, Germany (ethics vote No. 176/17).

## Results

We retrospectively evaluated the data of 109 refugees, 819 adult control ICU patients and 224 pediatric control ICU patients. 69.7% (n=76/109) of refugee patients, 66.7% (n=546/819) of control adult ICU and 62.1% (n=139/224) of pediatric patients were male. The median age of refugee, control adult ICU patients and control pediatric ICU patients was 16 years with standard deviation (SD) 14 years, 66 years (SD: 15.5 years), and 4 years (SD: 6.4 years), respectively. The refugees’ most frequently reported countries of origin were Afghanistan (34.9%, n=38), Syria (17.4%, n=19) as well as Ethiopia, Eritrea and Iran (8.3% each, n=9, Table 1 (Tab. 1)). Refugee patients were most frequently admitted to the Department for Pediatrics (n=58/109; 53.2%).

Data regarding duration of stay in Germany were available for n=95/109 (87.2%) of refugee patients. The distribution according to time-frame was ≤3 months n=29/95 (30.5%), >3 to <6 months n=14/95 (14.7%), ≥6 to <12 months n=15/95 (15.8%), ≥12 to <18 months n=23/95 (24.2%) and ≥18 months with n=14/95 (14.7%). 

The prevalence of at least one MDRGN in refugee and control adult ICU patients was n=45/109 (41.3%; 31.9–51.1) and n=47/819 (5.7%; 4.2–7.6), respectively (p<0.05; Table 2 (Tab. 2)). In control pediatric ICU patients, n=20/224 (8.9% [5.5–13.5]; p<0.05; Table 2 (Tab. 2)) were found to be positive for at least on MDRGN.

Of n=45 refugees testing positive for MDRGN, three individuals tested positive for two MDRGN species each, n=2 with *Escherichia coli*, (resistance due to extended spectrum β-lactamase [ESBL] and additional resistance to fluoroquinolones (*E. coli* ESBL/FQ) + *Klebsiella pneumoniae* ESBL, each), n=1 with *E. coli* ESBL + *Citrobacter freundii* with additional resistance to 3^rd^ generation cephalosporins and fluoroquinolones (*C. freundii* Ceph/FQ), resulting in n=48 MDRGN isolates found in n=45 refugees. As also given in Table 2 (Tab. 2), of n=819 control ICU patients, four individuals tested positive for two MDRGN species each, n=1 with *E. coli* ESBL + *K. pneumoniae* ESBL/FQ; n=1 with *E. coli* ESBL + *K. pneumoniae* ESBL; n=1 with *K. pneumoniae* ESBL/FQ + *Pseudomonas aeruginosa* with resistance to piperacillin, any 3^rd^/4^th^ generation cephalosporin, fluoroquinolones, and carbapenems (*P. aeruginosa* Pip/Ceph/FQ/CR); n=1 with *E. coli* ESBL + *Morganella morganii* Ceph/FQ. Concerning all MDRGN species, the most frequently detected MDRGN in refugee and adult control ICU patients were *E. coli* ESBL/FQ (n=25/51; 49.0%; 34.8–63.4) and *E. coli* ESBL (n=22/48; 45.8%; 31.4–60.8), respectively. *Enterobacteriacae* or *Acinetobacter baumannii* expressing CR were found neither in refugees nor in control ICU patients. As also given by Table 2 (Tab. 2), only one pediatric ICU patient tested positive for two MDRGN species (n=1 with *E. coli* ESBL + *K. pneumoniae* ESBL). In pediatric patients, the most frequently detected MDRGN was *E. coli* ESBL (n=9/21; 42.9; 21.8–66.0).

Concerning MRSA, n=20/109 refugee (18.3%; 11.6–26.9) and n=4/819 (0.5%; 0.2–1.2) control ICU patients tested positive. In control pediatric ICU patients, n=4/224 (1.8%; 0.5–4.5) tested positive for MRSA.

**Figure 1:**
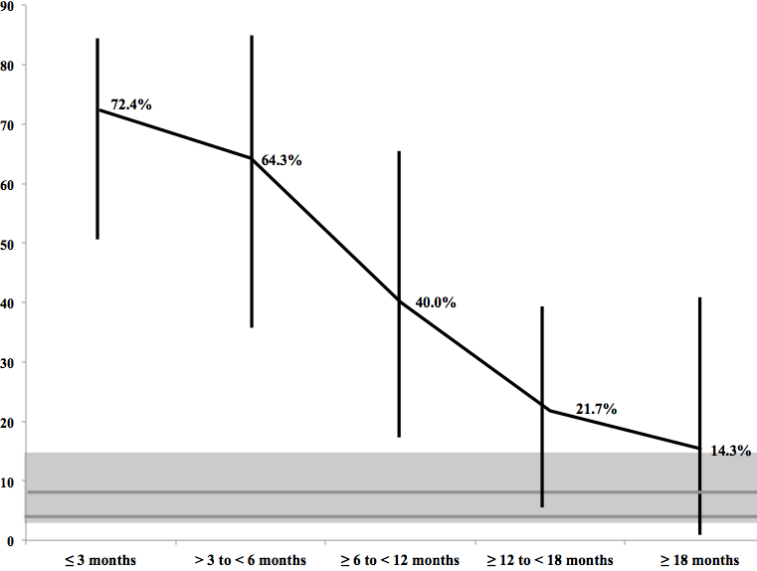
Prevalence of multidrug-resistant Gram-negative organisms (MDRGN) in refugee patients by their reported time since arrival in Germany (black), MDRGN prevalence in critically ill control adult patients (lower grey line) and pediatric control patients (upper grey line). Light grey shading: 95% confidence interval for the controls’ MDRGN prevalence in total.

MDRGN prevalence in refugees seems to decline according to refugees’ duration of stay since arrival in Germany. With regard to all detected isolates, the highest MDRGN prevalence was observed in refugees who had been in Germany ≤3 months (72.4%; 52.8–87.3), significantly exceeding the MDRGN prevalence in control adult ICU patients (5.7%; 4.2–7.6) as well as pediatric ICU patients (8.9%; 5.5–13.5) by almost 13-fold and 8-fold, respectively. MDRGN prevalence further decreased to 64.3% (35.1–87.2; arrived >3 to <6 months ago) and 40.0% (16.3–67.7; =6 to <12 months). If the duration of stay in Germany was ≥12 to <18 months, the prevalence significantly differed from control adult ICU patients later on, with 21.7% (7.5–43.7; ≥12 to <18 months; p=0.01). However, if duration the duration of stay was ≥18 months, no significant difference was observed (14.3%; 1.8–42.8; p=0.2; Figure 1 (Fig. 1)), also compared to the pediatric group (8.9%; 5.5–13.5; p=0.63).

MRSA prevalence in refugees was initially 31.0% (15.3–50.8; duration in Germany ≤3 months) and later 14.3% (1.8–42.8; ≥18 months); it was significantly higher than in control adult ICU patients (0.5%; 0.1–1.2). For control pediatric ICU patients, the difference is significant initially (1.8%; 0.5–4.5; p<0.0001). High significance was also observed when compared to refugees who arrived ≥18 months ago (p=0.04). Due to the small case number, the dynamics such as demonstrated for MDRGN were not reliably evaluable.

## Discussion

Refugee patients have previously been shown to be at significant and inherent risk of carrying MDRGN or MRSA [1], [2], [3], [4], [5], [6], [7], [8]. The need for stringent infection control management for refugee patients, e.g., screening procedures for MDRO, has therefore been discussed for German hospital settings [1], [2], [3], [4], [5], [20]. Stringent infection control management for refugee patients has been implemented at the University Hospital Frankfurt, Germany, since June 2015, consisting of screening for MDRO on the day of admission, pre-emptive isolation and release from isolation as soon as negative results for both MDRGN and MRSA are available [1].

Several studies have shown that median duration of MDRGN colonization was up to one year [9], [21], [22]. However, up to now, it had not yet been investigated whether MDRO prevalence in refugees approaches MDRO prevalence in non-refugee patients, which is essential for establishing appropriate infection control management.

Having mainly arrived from Afghanistan and Syria (Table 1 (Tab. 1)) ≤3 months ago, refugees’ overall prevalence of MDRGN and MRSA amounted to 41.3% and 18.3% (Table 2 (Tab. 2)), respectively. Both findings are consistent with previous findings on the countries of origin and overall prevalence of MDRO in refugee patients in Germany published in 2016 and 2017 [1], [2], [3], [4], [5]. Our findings on the refugees’ most frequently reported countries of origin broadly agree with data from the German Federal Office for Migration and Refugees, which reports Syria, Iraq and Afghanistan as the most frequent countries of origin [23], [24].

Interestingly, MDRGN prevalence in refugee patients seems to depend on the time elapsed since arrival in Germany (Figure 1 (Fig. 1)): the highest MDRGN prevalence was found in refugees who recently arrived in Germany (72.4%), exceeding the adult controls’ MDRGN prevalence by almost 13-fold. Gradually declining, MDRGN prevalence in refugee patients reached that of control adult and pediatric ICU patients at the earliest in the group who arrived ≥18 months ago. At this point, however, absolute MDRGN prevalence in refugee patients still exceeded the adult non-refugee patients by almost 3-fold. Considering that the refugee cohort consisted of 109 individuals, while that of the critically ill adult patients was >800 individuals and critically ill pediatric patients was >220 individuals, the widths of the groups’ overall 95% CI vary greatly. Since the critically ill adult patients’ 95% CI is narrow (around 3%), the 95% CI in the refugee group contributes about 20% just by itself. A similar effect is given when compared to the pediatric patients, where the width of the 95% CI is around 7%. Furthermore, since the refugee subgroups consist of only around 20 individuals each, the 95% CI becomes even wider, e.g., for the group who had been in Germany >18 months, it was around 35%. Therefore, the non-significance in our study needs to be carefully discussed. Furthermore, it shows that future settings on even larger refugee cohorts are warranted to verify the dynamics and significances observed in this pilot investigation. Nonetheless, these findings could justify applying specific infection control procedures to refugees whose duration of stay in Germany is less than 18 months.

Concerning the guidelines of infection prevention commission (KRINKO) at the Robert Koch Institute, isolation is recommended for all hospitalized patients testing positive for MRSA or carbapenem-resistant MDRGN, and additionally for MDRGN resistance to 3^rd^/4^th^ generation cephalosporins and fluoroquinolones, when such patients are admitted to any ICU or intermediate care unit [10], [11]. In our study, isolation regimens would therefore apply to 31.2% of refugee patients overall. This value is almost identical to our observations in a refugee patient cohort investigated in 2015 [1]. Since data from other studies (e.g. Heudorf et al. also from Germany, Ravensbergen et al. from The Netherlands or Angeletti et al. from Italy [1], [2], [3], [4], [25], [26]), found MRSA and MDRGN rates similar to those published by the present authors in 2016 and 2017, we expect similarly high rates of refugee patients who may also require isolation. In contrast, isolation would apply only to 4.0% of critically ill adult control patients overall. The fact that isolation measures would apply to 26.1% of refugee patients having arrived ≥12 to <18 months ago (this rate exceeds that of the adult controls by more than 6-fold) additionally supports our suggestion that specific infection control procedures for refugees might be necessary if they have been in Germany for less than 18 months.

Regarding MRSA prevalence, we found that 19.3% (12.3–27.9), 0.5% (0.1–1.2) and 1.8 % (0.5–4.5) of refugees, control adults and pediatric patients, respectively, tested positive. Even though the number of cases is too low to make a sound statement on epidemiological trends over time for MRSA, some *spa* types detected in refugees, e.g. t2251 or t5168, are dramatically less frequent in Germany (frequency around 0.01% each) but have often been described from South Africa and Sweden [27]. *Spa* type t304, which was found in five cases, is less frequent as well (0.42%), and has been described from Northern Europe (e.g. Norway, Sweden or Iceland) [27]. In contrast, spa types detected in control ICU patients (e.g. t002 or t127) are amongst the most common types in Germany, at rates of 6.9% and 2.4%, respectively [27]. Even *spa* type t003, which was found in one pediatric patient, is known to represent a very frequent (8.85%) Rhine Hesse MRSA subclone [27].

Although this pilot study demonstrates that refugees’ MDRGN prevalence depends on the duration of their stay in Germany, our data have some limitations. Data concerning medical history or antibiotic pre-treatment in refugees’ respective countries of origin were not available due to language barriers. refugees were formerly assumed to be in good medical condition [2]. However, they completely differ from medical tourists not only from an epidemiological point of view but also regarding the groups’ assumed health condition. The diversity of MDRGN found in medical tourists is different from that in refugees [2], and medical tourists are supposed to be ill. 

Additionally, the refugees’ living quarters might be relevant, since refugees living in smaller facilities might have a different risk of carrying MDRO than those living in mass camps. For future investigations, the refugee patients’ antibiotic pre-treatment, their entire medical history as well as their residential status might yield information that would provide deeper insight into their medical condition, which also have bearing on their MDRO prevalence.

Due to language barriers, the reliability with which a refugee’s exact time of arrival in Germany is reported may remain unclear in some cases. Moreover, this study only documented refugees’ duration of stay in Germany, and did not take time spent in other European countries into account. Their date of arrival in Germany is very likely preceded by different durations spent en route and by different routes taken [28], [29]. The term “time since arrival in Germany” is therefore a tool which helps define a common denominator. Since this term disregards the individual’s history, future investigations should attempt to include more such details. We feel that this could contribute to the discussion on targeted infection control for refugee patients. 

However, it must be mentioned that our findings are consistent with O’Fallon et al., who reported that the clearence of MDRGN can range from a few weeks up to one year [9]. We therefore assume that the impact of possible imprecision in the exact duration of stay since arrival in Germany is rather small.

Concerning the median age of refugees (16 years) compared to the median age of adult and pediatric controls (66 years and 4 years, respectively), both control groups do not match optimally. An adolescent control group matched by age and gender would have been most suitable,but the transition from childhood to adolescence is difficult to categorize and the group size in this study was too small to generate a cohort with statistically sound sample design and size. 

In addition, follow-up data for the individual patients were missing in this pilot study. This might be important to evaluate the respective individual’s MDRGN dynamics. Since we have chose a retrospective design, only an epidemiological “snapshot” is given. This could be inferior to a prospective setting that could give extended insight in the epidemiological dynamics of MDRO in refugee patients. However, an evaluation with a prospective approach could be too difficult due to refugees’ mobility within Germany or even within the European Union. As reported by the Federal Office for Migration and Refugees (Bundesamt für Migration und Flüchtlinge, BAMF), refugees are registered at a reception center after their arrival in Germany [30]. Since registration is followed by distribution among the German Federal States, refugees’ long-term place of residence may be far from the initial registration facility. In terms of a prospective follow-up setting, refugees would therefore not be available for later analysis. This, in turn, hampers prospective data acquisition. In future studies, it may also be pertinent to compare the MDRGN prevalence in refugees to that of recognized migrants. Unfortunately, these terms are often used interchangeably, even though a “refugee” is clearly, legally defined under The 1951 Refugee Convention by the United Nations High Commissioner for Refugees (UNHCR) [31] as “someone who is unable or unwilling to return to their country of origin owing to a well-founded fear of being persecuted for reasons of race, religion, nationality, membership of a particular social group, or political opinion.” [31]. In contrast, migrants are defined by the UNHCR as persons who “choose to move not because of a direct threat of persecution or death, but mainly to improve their lives by finding work, or in some cases for education, family reunion, or other reasons. Unlike refugees who cannot safely return home, migrants face no such impediment to return.“ [32].

Due to the fact that refugees with unknown durations of stay in Germany still need to be managed in terms of stringent infection control, we decided not to exclude them from this investigation. Regarding the generally high MDRGN prevalence in refugee patients, we recommend that these patients be managed as refugee patients having arrived <18 months ago.

## Conclusions

This pilot study addressed the MDRO prevalence in refugee patients depending on their duration of stay in Germany. MDRGN prevalence in refugees seems to decline over time since their arrival in Germany. 18 months or more after refugee patients’ arrival in Germany, their MDRGN prevalence no longer significantly differs from the controls’ MDRGN prevalence any longer. In contrast, MRSA prevalence seems to remain almost stable over time. 

18 months after their arrival, refugees’ and locals’ MDRGN prevalence are statitically similar, although that of the refugees is still elevated. MRSA prevalence, however, was still 14% even 18 months after their arrival, which exceeded that of non-refugees by almost 8-fold. This might indicate that specific infection control management and precautionary measures also need to be considered for a longer time span after arrival. For refugee patients having arrived <18 months ago, specific infection control management and precautionary measures remain necessary in hospital settings.

## Notes

### Acknowledgements

Volkhard A. J. Kempf was supported by a grant from the Deutsche Forschungsgemeinschaft (DFG research unit 2251). The authors received no specific funding for this work.

### Competing interests

The authors declare that they have no competing interests.

## Figures and Tables

**Table 1:**
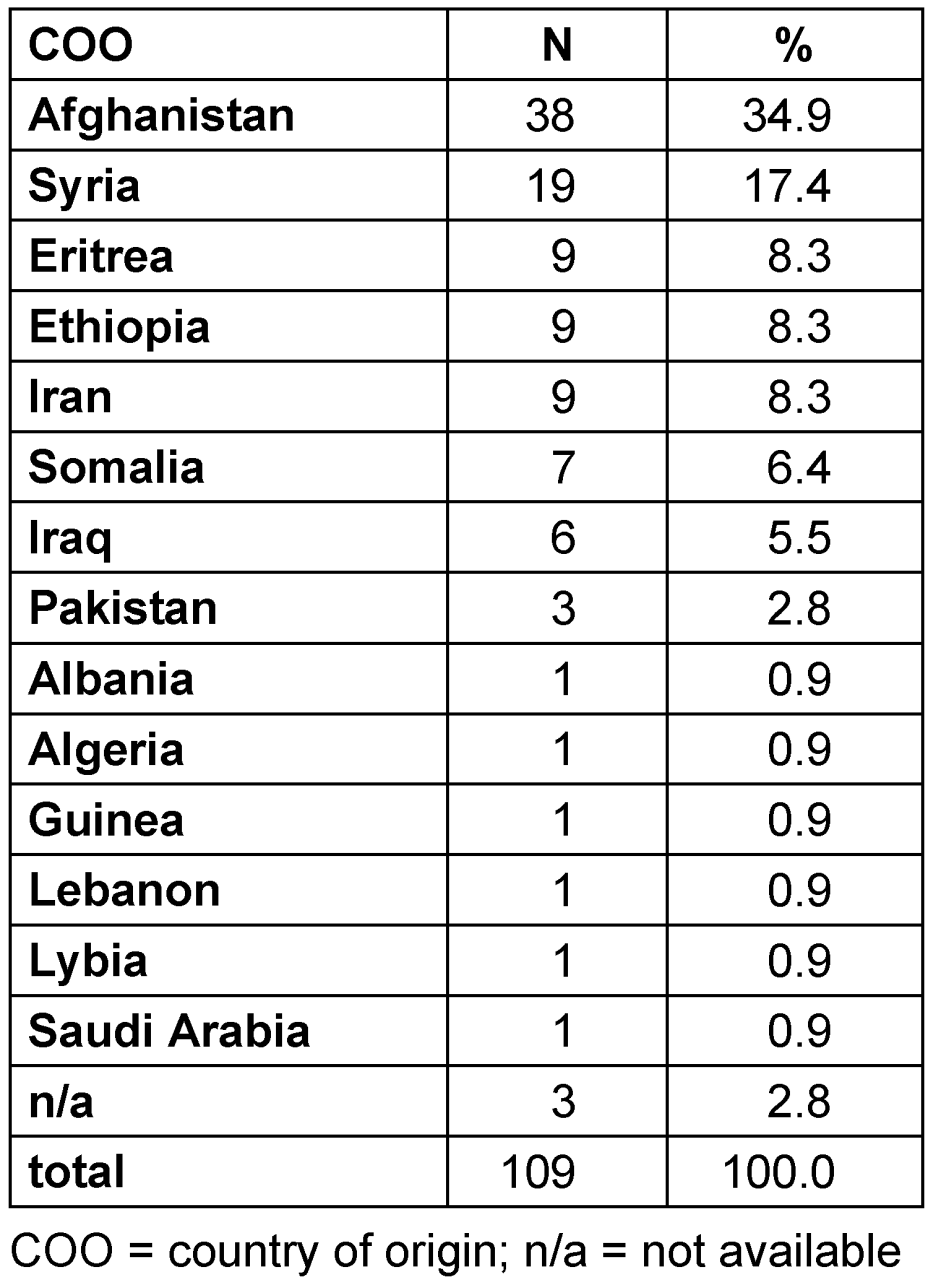
Refugees’ countries of origin

**Table 2:**
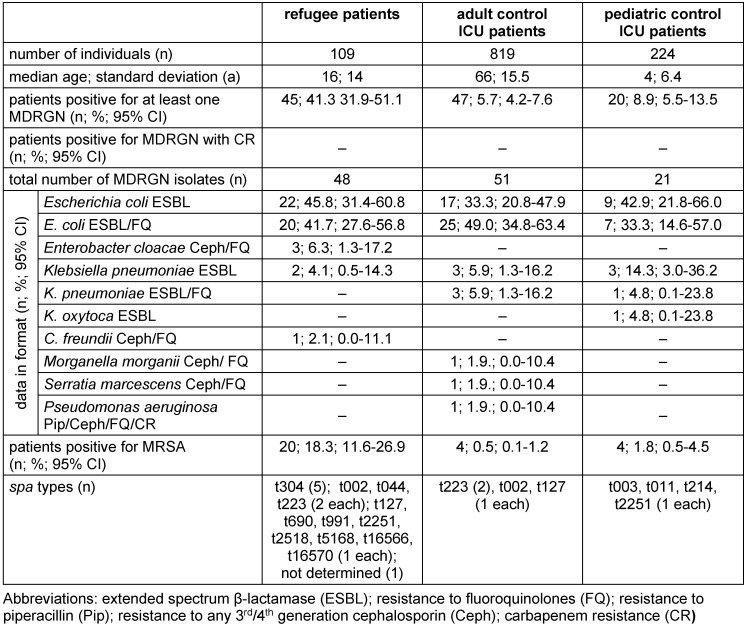
Prevalence of multidrug-resistant Gram-negative organisms (MDRGN) and methicillin-resistant *Staphylococcus aureus* (MRSA) in refugee and control patients admitted to the intensive care unit (ICU).
